# An Unusual Case of Systemic Lupus Erythematosus and Hemophagocytic Syndrome

**DOI:** 10.1155/2016/8957690

**Published:** 2016-02-14

**Authors:** Saika Sharmeen, Nazia Hussain

**Affiliations:** ^1^Department of Medicine, St. Luke's-Roosevelt Hospital Center, New York, NY 10025, USA; ^2^Department of Medicine, Icahn School of Medicine at Mount Sinai, New York, NY 10025, USA; ^3^Division of Rheumatology, Department of Medicine, St. Luke's-Roosevelt Hospital Center, New York, NY 10025, USA

## Abstract

Hemophagocytic syndrome (HS) or hemophagocytic lymphohistiocytosis (HLH) is an immune mediated phenomenon that can occur in the setting of an autoimmune disease, chronic immunosuppression, malignancy, or infection. It has been more commonly described in the pediatric population and less commonly in adults. We describe a case of a 52-year-old male who presented with a rash. He simultaneously met the Systemic Lupus International Collaborating Clinics (SLICC) criteria for the diagnosis of systemic lupus erythematosus (SLE) and the diagnostic criteria of HS as described in the hemophagocytic lymphohistiocytosis (HLH) 2004 trial. The bone marrow on autopsy showed the presence of abundant hemosiderophages with focal hemophagocytosis. SLE-associated HS might be underdiagnosed due to the overlap in clinical findings. This case represents the importance of prompt diagnosis and treatment of such a potentially fatal clinical syndrome.

## 1. Introduction

Systemic lupus erythematosus (SLE) is one of the collagen vascular diseases which may induce hemophagocytic syndrome (HS). The etiology of HS is essentially unknown but is thought to result from uncontrolled T lymphocyte activation that leads to macrophage activation and an increment of some cytokines such as tumor necrosis factor-*α*, interleukin-1 (IL-1), IL-6, IL-18, and interferon-*γ* [1]. Morphologically benign hemophagocytic histiocytes infiltrate the bone marrow and various organs, including the lymph nodes, liver, and spleen. Patients usually present with an acute febrile illness, which can sometimes become fulminant and lethal. Common manifestations include high fever, pancytopenia, hepatosplenomegaly, elevated liver enzymes, and high blood triglyceride and ferritin levels. Coagulopathy and central nervous system dysfunction often ensue, and less frequently the lungs and cardiac tissues are involved [2]. SLE in conjunction with HS is a rare presentation that has mainly been described in case reports and retrospective studies. It is a rare presentation in male patients and even more difficult to diagnose in a patient with history of alcohol abuse or cirrhosis where coagulopathy, elevated liver enzymes, and hepatosplenomegaly may also be expected. We demonstrate this difficulty in this case report of an African American male with history of alcohol abuse found to have SLE and HS. To our knowledge such a case in this demographic has never been reported.

## 2. Case

A 52-year-old African American male with past medical history only significant for alcohol abuse presented with four days of a pruritic rash. He was in his usual state of health until four days prior to admission when he noticed the rash. He could not remember if he first noticed it on his chest, abdomen, or legs. Over the next two days, it became more pruritic. He denied any shortness of breath or tongue swelling, as well as any constitutional symptoms including subjective fevers, night sweats, chills, or weight loss. He admitted to not seeing a primary care doctor in two years. He also denied any gait disturbance, gastrointestinal complaints, or urinary complaints but noted that his urine has been darker than usual. He endorsed daily alcohol use of about two to three beers daily but denied smoking or illicit drug use. He worked for the state and was working as of one day prior to admission. He admitted to recent travel in the northeast region. He denied taking any over-the-counter drugs or herbal medications.

Vitals at presentation were as follows: temp 98.9 degrees Fahrenheit, RR 16/min, HR 113–117/min, and BP 160/80 mmHg, saturating 100% on room air. On physical exam, skin exam was significant for nonblanching purpura on anterior calf bilaterally, pruritic nonblanching macules, and papules on chest, back, arms, abdomen, and petechial rash on his palms and soles bilaterally. The rash spared the groin area. Oral, ocular, and genital mucosa was clear. There was no skin tenderness. There was mild mucosal bleeding on lower lip. His eye exam showed scleral icterus. He also had lymphadenopathy on neck exam. He denied ever having a similar rash. On abdominal exam, he was found to have hepatosplenomegaly, with abdominal distention, and normoactive bowel sounds. There was no fluid shift. There was moderate pitting edema bilaterally but no clubbing or cyanosis. Neurologic exam was unremarkable at presentation.

Labs on presentation (Table 1) were significant for thrombocytopenia, hyponatremia, bilirubinemia, leukocytosis with left shift, normocytic anemia, and coagulopathy with elevated INR. His urine toxicology was negative. Urinalysis showed protein, moderate bilirubin, more than 182 RBC, 13 WBC, and trace leukocyte esterase. Urine sediment showed many RBCs with less than 50% dysmorphic cells but no obvious casts.

EKG showed sinus tachycardia 105 bpm, with left axis deviation. Chest X-ray was unremarkable. Computed tomography (CT) of abdomen and pelvis with contrast showed multiple hypodense hypovascular lesions scattered throughout the liver causing surface contour deformity with numerous subcentimeter periportal, periaortic splenic, and periesophageal lymph nodes. Spleen was notable for linear as well as rounded ill-defined hypodensities suggestive of splenic infarcts, abscess, or malignancy. The initial differential was broad including infectious etiology causing sepsis, or malignancy. Given the appearance of the rash as well as history of travel to northeast, the patient was started on doxycycline for suspicion of Rocky Mountain Spotted Fever and supportive management for his remaining findings.

Over the next few days of his hospital course he developed acute mental status change with CT head without contrast showing nonspecific left parietal/temporal scalp soft tissue swelling and no other pathologies. On hospital day 2 he was transferred to the medicine intensive care unit (ICU) for being febrile with *T* 101 degrees Fahrenheit, renal failure, hepatic dysfunction, and worsening coagulopathy. CT chest without contrast showed focal areas of ground glass attenuation right greater than left. These findings may represent in this clinical setting pulmonary hemorrhage as well as bilateral pleural effusions. All blood cultures, urine cultures, and all infectious workup were negative (Table 3). In the ICU he had a cardiac arrest requiring cardiac resuscitation and intubation, with return of spontaneous circulation. His hospital course was complicated with persistent fevers, gastrointestinal bleeding, acute kidney injury requiring hemodialysis, hypotension requiring pressor, and eventual acute respiratory distress syndrome with worsening liver failure. Later the lab results showed elevated ferritin level and LDH (Table 2). His rheumatologic workup (Table 3) revealed high titers of antidouble stranded DNA with low complement (C3/C4). Antinuclear antibody (ANA) was positive, antihistone antibodies (ab) negative, anti-Smith ab negative, antiribosomal P ab negative, antismooth muscle ab negative, and cardiolipin IgG/IgM moderately elevated. The patient was initially treated with broad spectrum antibiotics, despite negative cultures and infectious workup.

He received supportive management with packed red blood cell transfusion, fresh frozen plasma (FFP), and platelets. He was also treated with pulse dose steroids for 3 days and then intravenous (IV) Medrol 40 mg + 30 mg daily. He also received plasmapheresis followed by IV Cytoxan with mild initial improvement, but unfortunately his condition deteriorated and the patient passed away. The autopsy revealed marked diffuse intra-alveolar hemorrhage, pulmonary edema, cirrhotic liver with cholestasis, acute tubular necrosis, chronic pancreatitis, infarcted spleen, and evidence of esophageal varices in a patient with known chronic alcoholism. The bone marrow showed the presence of abundant hemosiderophages with focal hemophagocytosis, which added to the presence of fever, splenomegaly, skin rash, cytopenia, and increased ferritin which met the criteria for hemophagocytic lymphohistiocytosis.

## 3. Discussion

Our patient met 4 out of 17 (including at least one clinical criterion and one immunologic criterion) of the Systemic Lupus International Collaborating Clinics (SLICC) criteria for the diagnosis of SLE [3]. Our patient's findings included thrombocytopenia and hemolytic anemia as two clinical criteria and positive anti-dsDNA ab and low complement (C3/C4) as immunologic criteria. Our patient also met the diagnostic criteria for HS as described in the hemophagocytic lymphohistiocytosis (HLH) 2004 trial.

He had a fever of 38.5°C for 7 days or more, splenomegaly, hemoglobin of 9 g/dL or less, platelets less than 100, hyperferritinemia, and hemophagocytosis in bone marrow [4]. Lambotte et al. described 8 cases where SLE was diagnosed simultaneously with HS pancytopenia with high ferritin level, uncommon in SLE, which is highly suggestive of HS [5]. Parodi et al. proposed preliminary diagnostic criteria for macrophage activation syndrome as a complication of juvenile SLE. The diagnostic criteria have a sensitivity and specificity of 92.1% and 90.9%, respectively, and an OR of 116.7% with a confidence interval between 21.9 and 621.6 at 95% confidence [6]. According to the diagnostic criteria proposed by Parodi et al., our patient met all clinical criteria including fever (>38°C), hepatomegaly (≥3 cm below the rib margin), splenomegaly (≥3 cm below the rib margin), hemorrhagic manifestations (purpura, easy bruising, or bleeding gums), and dysfunction of the central nervous system (irritability, disorientation, lethargy, headache, convulsions, or coma). He also met most of the laboratory criteria including cytopenia in 2 or more cell lines [4 leukocytes × 109/L, hemoglobin 90 g/L, or platelets 150 × 109, increased aspartate aminotransferase (>40 units/L), increased lactate dehydrogenase (>567 units/L), hypofibrinogenemia (fibrinogen <1.5 g/L), and hyperferritinemia (ferritin >500 *μ*g/L)]. The histopathologic criteria were also met by evidence of hemophagocytic macrophages in bone marrow. For diagnosis, the simultaneous presence of at least one clinical criterion and at least 2 laboratory criteria is required.

HIV, cytomegalovirus, and Epstein-Barr virus are among the reported viral causes of HS [7, 8]. Although infections are a possible trigger for HS, our patient had an extensive infectious workup which was all shown to be negative (Table 2). Unfortunately, liver biopsy could not be done due to the coagulopathy. In addition, his disease continued to progress despite broad spectrum antibiotics and supportive treatment. His mild clinical improvement after pulse steroids and IV Cytoxan was in favor of the trigger for HS being autoimmune in etiology. This patient also received plasmapheresis without improvement. The presence of chronic alcoholism causing cirrhosis further complicated the diagnosis.

The current literature suggests that the goal of therapy for patients with HLH is to suppress life-threatening inflammation by destroying immune cells. The first treatment protocol for HLH (HLH-94) consists of induction therapy with weekly treatments of dexamethasone and etoposide (VP-16), followed by cyclosporine. Intrathecal methotrexate is given to those with central nervous system disease. After induction, patients who are recovering are weaned off therapy, while those who are not improving are continued on therapy as a bridge to allogeneic hematopoietic cell transplantation [9]. The newer HLH protocol initiated in 2004 (HLH-2004) differs from HLH-94 by earlier use of cyclosporine during the induction phase of treatment and adds hydrocortisone to intrathecal methotrexate. Treatment of secondary HLH is directed at control of the underlying condition. If unsuccessful, cytotoxic agents such as those in HLH-2004, steroids, intravenous *γ*-globulin, or targeted immune therapy have been used [10]. Steroids and other immunosuppressants including Rituximab have also been reported in successfully treating HS in the setting of SLE [11, 12]. Wong et al. reported an incidence of SLE-associated HS of 6 cases during a 3.5-year period among 250 SLE patients. They alluded to the fact that SLE-associated HS might be underdiagnosed due to the overlap in clinical findings [13]. Moreover, this case was even more difficult to diagnose because many of the findings that would be expected in a patient with alcoholic cirrhosis were also present in this patient.

## 4. Conclusion

In conclusion, a prompt diagnosis is essential for treating HS in the setting of SLE. Clinicians should raise a high index of suspicion in such a case.

## Figures and Tables

**Table 1:** Lab values on presentation.

Variable	Lab values on admission	Reference range
Sodium	118	136–146 mmol/L
Potassium	4.9	3.5–5.1 mmol/L
Chloride	87	96–107 mmol/L
Carbon dioxide	23	22–30 mmol/L
Blood urea nitrogen	10	8–24 mmol/dL
Creatinine	0.89	0.66–1.25 mg/dL
Glucose	101	74–106 mg/dL
eGFR	90	>90
Calcium	7.7	8.4–10.3 mg/dL
Corrected calcium^*∗*^	8.7	8.5–10.5 mg/dL
Protein, total	8.7	6.3–8.2 g/dL
Albumin	2.7	3.5–5 g/dL
Bilirubin, total	4.8	0.2–1.3 mg/dL
Bilirubin, direct	3.5	0.0–0.4 mg/dL
ALP	187	38–126 U/L
AST	366	15–46 U/L
ALT	66	13–69 U/L
WBC	14.3	3.4–11 k/*μ*L
Hemoglobin	8.3	13.0–17 g/dL
Hct	27.7	38–51%
Platelet	72	150–450 k/*μ*L
MCV	90.7	80–100 fL
Band	23	
Eosinophils (%)	2	0.0–0.6%
Neutrophil (%)	70	40–74%
Lymphocytes (%)	3	18–44%
Monocytes (%)	2	4.7–12.0%
Basophil (%)	0	0.1–1.4%
Partial thromboplastin time	51.3	22.5–35.5 secs
INR	3	0.9–1.1
PT	30.8	11.8–14.3 secs

^*∗*^The normal albumin level defaults to 4 mg/dL standard units.

**Table 2:** Additional labs.

Variable	Measurement	Reference range
DAT broad spectrum Coombs Ser (Direct Coombs)	Negative	Negative
GGT	100	12–58 U/L
Tryptase	4	2–10 ng/mL
Lipase	22	23–300 U/L
Uric acid	7.1	3.5–8.5 mg/dL
Lactic acid	7.4	0.7–2.1 mmol/L
Ammonia	42	9–30 *μ*mol/L
Cryoglobulin	Negative	Negative
Creatine kinase	920	55–170 U/L
CRP, high sensitivity	10.4	Null
Sedimentation rate	100	0–13 mm/hr
LDH	2387	313–618 U/L
ADAMTS13 Activity	84	68–163% activity
G6PD	Adequate	Adequate
Haptoglobin, quant	22	30–200 mg/dL
Ferritin	700.7	22.0–322.0 ng/mL
Iron	41	49–181 *μ*g/dL
Iron binding capacity, total (TIBC)	265	261–462 *μ*g/dL
Thyroid stim. hormone	1	0.55–4.78 mIU/mL
Cortisol AM	16.54	4.0–22.0 *μ*g/dL
Factor XI assay	66	50–150%
Factor X assay	66	50–150%
Factor II activity	64	70–150%
Factor VIII assay	279	50–150%
Factor VII assay	16	50–150%
Factor V assay	49	50–150%
Fibrinogen	123	225–483 mg/dL
D-dimer	13.29	<0.5 *μ*g/mL

**Table 3:** Autoimmune and infectious workup.

Variable	Measurement	Reference range
ANA	Positive	Negative
ANA, quantitative	1 : 160	Negative
ANA, pattern	Speckled	Negative
ds DNA ab IgG Titer	300 or greater	0–29 IU/ML
C3	23	90–180 mg/dL
C4	3	10–40 mg/dL
Anti-Smith (Sm) interp.	Negative	Negative
SM-RNP antibodies interp.	Negative	Negative
SM-RNP ab interp.	Negative	Negative
Smith (ENA) ab, IgG	0.8	<16.00 EU/mL
SSA (Sjogren ab)	0.7	<16.00 EU/mL
SSB (Sjogren ab)	1	<16.00 EU/mL
Ribosomal P ab	<1.0	Negative
Smooth muscle ab	Negative	Negative
LKM 1 ab IgG	<20.0 U	Negative
GBM ab IgG	<1.0	<1.0 AI no antibody detected
Histone ab	1.2	<1.0 U negative
Cardiolipin ab IgM	24	Low to medium positive
Cardiolipin ab IgG	20	Indeterminate
*β*2-Glycoprotein I IgM	Negative	Negative
*β*2-Glycoprotein I IgA	9	Negative
ANCA vasculitides		
Proteinase 3 ab	<1.0	<1.0
Myeloperoxidase ab	<1.0	<1.0
Hep A ab, IgM	Nonreactive	Nonreactive
Hep A ab, total	Nonreactive	Nonreactive
Hep B sAg	Negative	Negative
Hep B Core ab, total	Reactive	Nonreactive
Hep BS ab	Reactive	Nonreactive
Hep C ab	Negative	Negative
HIV 1/2 ab screen, rapid	Nonreactive	Nonreactive
Cytomegalovirus, IgM	0.7	<0.9
Cytomegalovirus, Igg	Negative	Negative
EBV, DNA, QN, and PR-PCR	<200 COPIES/ML	Negative
Syphilis Screen Titer	Nonreactive	Nonreactive
Lyme Disease ab Screen	0.58	<0.91
RMSF IgM and IgG	Not detected	Not detected
*B. Quintana* IgM Scr	Negative	Negative
*B. Quintana* IgG Scr	Negative	Negative
*B. Henselae* ab IgG	Negative	Negative
*Brucella* IgG	0.16	<0.80 antibody not detected
*Brucella* IgM	0.07	<0.80 antibody not detected
AFB culture	Negative	Negative
Fungal culture	Negative	Negative
Viral culture	No virus isolated after 4 days	Negative
Gram stain	Few 2+ PMN no organisms	Negative
Bacterial culture	No growth	Negative
